# A Systematic Review of Conservatively Managed Isolated Extra-Articular Proximal Phalanx Finger Fractures in Adults

**DOI:** 10.1016/j.jpra.2024.05.002

**Published:** 2024-05-09

**Authors:** Min Zhang, Melissa Hirth, Tanya Cole, John Hew, Pelicia Lim, Sally Ng

**Affiliations:** a Plastic and Reconstructive Surgery Department, Austin Health, 145 Studley Rd, Heidelberg, VIC 3084, Australia; b Occupational Therapy Department, Austin Health, 145 Studley Rd, Heidelberg, VIC 3084, Australia; c Malvern Hand Therapy, 253 Wattletree Road, Malvern, VIC 3144, Australia

**Keywords:** Conservative management, Finger injuries, Phalangeal fractures, Orthotic Devices, Splint

## Abstract

**Study design:**

Systematic review

**Background:**

Proximal phalangeal fractures of the hand are challenging to treat, and significantly impact hand function and quality of life if poorly managed.

**Purpose:**

A systematic review to determine the efficacy of conservatively managed extra-articular proximal phalanx fractures to optimise recovery and prevent the need for surgical intervention and its associated risks.

**Methods:**

A literature search that included variations of the phrases ‘proximal phalanx’, ‘fracture’ and ‘conservative management’ was performed on 17 December 2023 using seven electronic databases and trial registries. Article screening, data extraction and critical appraisal using the Structured Effectiveness Quality Evaluation scale was performed independently.

**Results:**

Seven studies that captured 389 fractures from 356 unique patients were included. Studies were of level II to IV evidence and included one comparative cohort study and six prospective case series. Interventions involved timely rehabilitation, a plaster or orthotic device, controlled metacarpophalangeal joint flexion and free mobilisation of the interphalangeal joints. A weighted mean total active motion score of 249° was achieved, with 99.5% (387/389) of fractures achieving union.

**Conclusions:**

This systematic review cautions against definitive recommendations on conservative techniques for managing proximal phalanx fractures due to limitations of the available literature. However, our findings tentatively supports non-operative approaches as an alternative to surgery.

## Introduction

Fractures of the proximal phalanx (P1) constitute approximately 20% of all finger fractures,^1^ and pose a unique challenge in clinical management.^2^ If improperly addressed, these fractures can have a profound impact on hand function and overall quality of life. The complexities commonly arise from proximal interphalangeal joint (PIPJ) stiffness, often attributed to tendon adhesion and skeletal deformity. Displaced P1 fractures present distinctive challenges, manifesting as palmar angulation owing to interossei muscle insertion at the P1 volar base and hyperextension of the distal fragment from the central slip acting on the middle phalanx base.^3^^,^^4^ Given the crucial role of the P1 volar shaft surface in forming the flexor tendon sheath floor, achieving anatomical reduction becomes paramount for optimising flexor tendon glide.

The goal of P1 fracture management is to achieve a well-aligned, pain-free and stable digit with good range of motion (ROM) to provide functional movement.^3^ This requires an early motion program that ensures tendon gliding and joint mobility, while providing skeletal stability through conservative or surgical means.

Conservative approaches stabilise fracture fragments using soft tissue and are recommended for stable fractures.^4^ Freeland et al.^3^ described an orthosis with dynamic finger positioning at rest or immobilised with the metacarpophalangeal joints (MCPJs) supported at 50-70° flexion and the PIPJ supported at 0-15° flexion to minimise joint contracture. This position also enables intrinsic muscle relaxation and the extensors to act as a tension band over the P1 for stability. Active motion further compresses the fracture site and stimulates periosteal callus formation to reinforce the injury site.^3^^,^^5^

In contrast, surgery is traditionally indicated for unstable fractures or if deformity recurs after closed reduction. This additional ‘planned injury’ risks secondary scar formation and devascularisation of fracture fragments,^3^ leading to tissue adhesion and joint contracture. Thus, the benefit of increased biomechanical stability through surgical incision, risk of stiffness and delayed healing need to be considered.

### Purpose of the study

Despite the proven outcomes of conservative P1 fracture management, surgical fixation remains commonly recommended in the literature, particularly in open or unstable fractures with or without intra-articular involvement, rotational deformity or significant displacement.^6^^,^^7^ This systematic review seeks to provide evidence that supports conservatively managed extra-articular P1 fractures to optimise recovery and mitigate the necessity for surgical intervention and its associated risks.

## Methods

A protocol was registered on the PROSPERO international prospective register of systematic reviews (CRD42021270244) and performed according to the Preferred Reporting Items of Systematic Reviews and Meta-Analysis Protocols statement.^8^

Articles were sourced from database inception until 17 December 2023 from MEDLINE, Embase, Emcare using Ovid; trial registries (World Health Organization International Clinical Trials Registry Platform, Australian New Zealand Clinical Trials Registry and ClinicalTrials.gov) and the Cochrane Central register of Controlled Trials, using their respective websites.

A search strategy was designed with a clinical librarian using phrases and Medical Subject Headings (MeSH) related to ’proximal phalanx‘, ‘fracture’ and ‘conservative management’. Variations of these terms were tailored for each database (Supplement Table 1). No study setting or publication status restrictions were imposed. Attempts were made to obtain translations for publications in languages other than English.

### Selection of studies

References were imported onto Covidence to filter duplicates and facilitate screening. Five reviewers (MZ, JH, MH, TC and SN) independently short-listed all studies by screening titles and abstracts according to the eligibility criteria (Table 1). Articles were excluded if they were unrelated, or an English version of the full text could not be obtained. Disagreements were resolved through group discussion with all five reviewers to finalise the selection.

**Table 1:** Eligibility Criteria.

Inclusion criteria	Exclusion criteria
**Participants** ■ Adults (≥ 18 years) with acute extra-articular P1 fractures of the hand ■ Closed fractures ■ Open fractures only associated with simple lacerations or isolated digital nerve injury ■ Mixed population studies were included if the data for the population of interest could be isolated	■ Intra-articular fractures ■ Thumb phalanx fractures ■ Middle or distal phalangeal finger fractures ■ Fractures presenting after two weeks

**Interventions** ■ Non-surgical treatment modalities, which includes (but is not limited to): ■ Fracture reduction or manipulation ■ Orthotic intervention ■ Taping or strapping ■ Mobilisation	■ Surgical management, which includes (but is not limited to): ■ Open reduction internal fixation (ORIF) techniques such as plate or screw fixation ■ Closed reduction internal fixation (CRIF) techniques such as K-wire fixation

**Study design** ■ Randomised controlled trials ■ Cohort studies ■ Case control studies ■ Case series (n ≥ 10)	■ Cadaveric studies ■ Biomechanical studies ■ Animal studies ■ Review articles ■ Study protocols ■ Case reports ■ Surveys ■ Editorials ■ Commentaries ■ Conference abstracts ■ Letters

Authors were contacted if the data necessary to assess eligibility according to the above criteria was missing in their manuscript. Articles were excluded if authors failed to reply after two attempts of contact.

### Data extraction and management

As per the Cochrane Handbook of Systematic Reviews of Interventions,^9^ three reviewers (MZ, JH and TC) independently gathered data on study design, demographics, fractures, intervention, follow-up and outcomes. The primary outcome measure of interest was mean post-intervention ROM of any included finger joint. Secondary outcome measures included fracture union, pain, satisfaction, grip and pinch strength, return-to-work data and complications.

Outcomes for mixed population studies were recalculated if the population of interest was distinguishable from the gross data set. The weighted mean for outcomes of interest was calculated by multiplying each value with its corresponding sample size, summing these products and then dividing by the total of the sample sizes. This information was tabulated using Microsoft Word.

Five reviewers (MZ, JH, MH, TC and SN) independently assessed the study quality of all short-listed articles using the Structured Effectiveness Quality Evaluation Scale (SEQES),^10^ which scores interventional studies from 0-48 using 24 criteria relating to objectives, study design, participants, intervention, outcomes, analysis and recommendations. Disagreements were resolved by discussion with all five reviewers. A meta-analysis was deemed inappropriate owing to study designs which were heterogenous and of low-moderate quality. A narrative synthesis is therefore presented. A treatment protocol was synthesised by consolidating the common intervention characteristics across all included studies.

## Results

**Figure 1:**
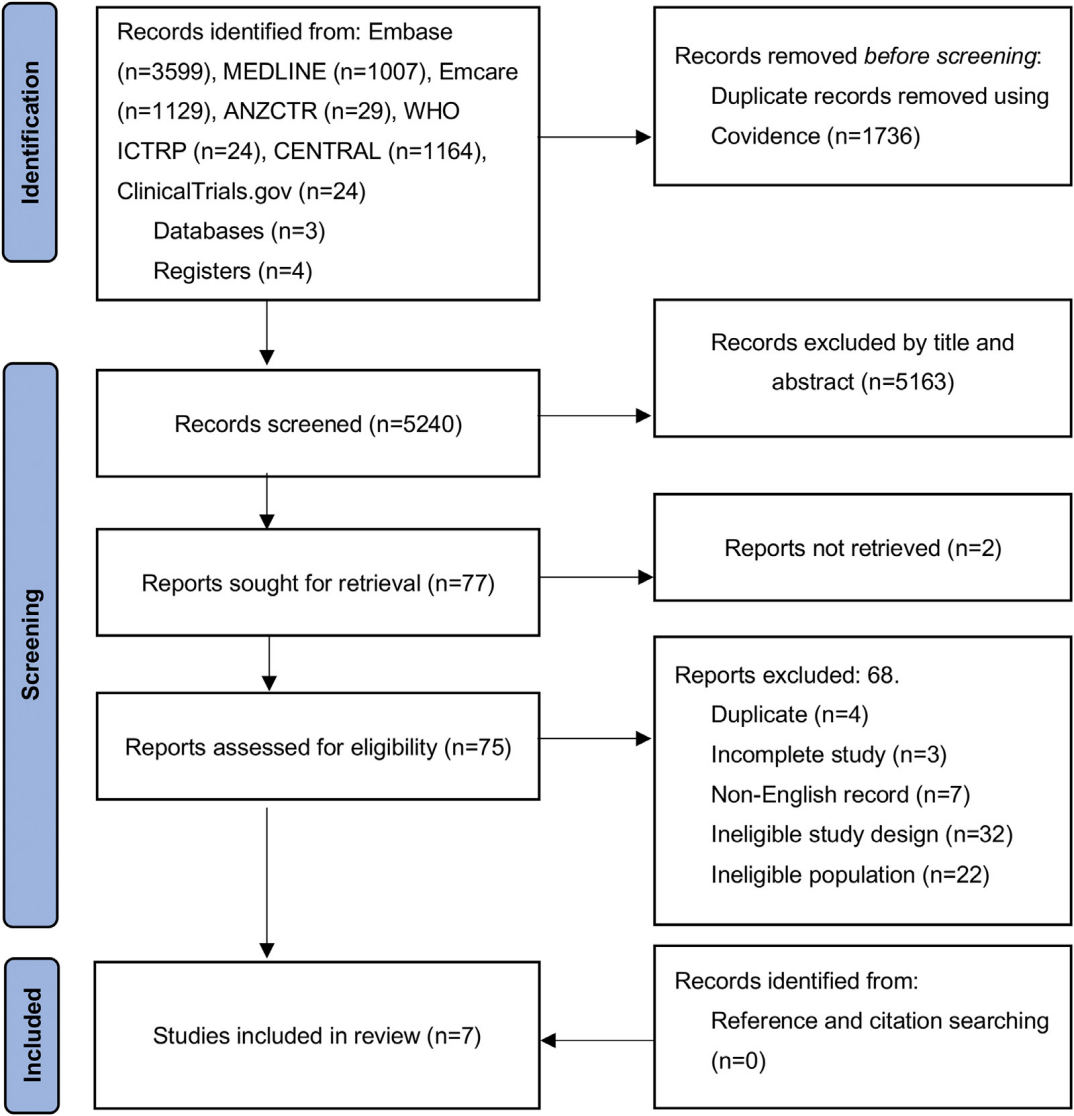
PRISMA Flow Diagram representing the search and selection of studies assessing the efficacy of conservative therapies for proximal phalanx fractures.

An extensive literature search was conducted which yielded seven studies^4^^,^^11^, ^12^, ^13^, ^14^, ^15^, ^16^ (Figure 1).

Study quality on the SEQES^10^ ranged from 14-33 out of a possible 48 (Table 2). One study^13^ was graded level II according to the Oxford Centre for Evidence-Based Medicine,^17^ as it used a randomised comparative approach. The remaining studies were case series and designated level IV.^4^^,^^11^^,^^12^^,^^14^, ^15^, ^16^

**Table 2:** Structured Effectiveness Quality Evaluation Scale (SEQES).^10^

	Descriptors	Study design							Subjects				Intervention			Outcome			Analysis					Recommendations	
	1	2	3	4	5	6	7	8	9	10	11	12	13	14	15	16	17	18	19	20	21	22	23	24	Total†
Thomine et al. (1983)^16^	1	0	0	1	0	1	1	0	0	1	0	2	1	1	0	1	1	1	0	0	0	2	1	1	16
Reyes and Latta (1987)^15^	1	0	1	1	0	1	1	0	1	1	0	0	1	1	0	1	1	1	0	1	0	0	1	1	15
Rajesh et al. (2007)^4^	1	0	0	1	0	1	1	0	1	1	0	2	1	1	0	1	1	1	0	0	0	1	0	0	24
Figl et al. (2011)^12^	1	0	0	1	0	1	1	0	0	0	0	2	1	0	0	1	1	1	0	1	0	1	1	1	14
Franz et al. (2012)^13^	2	2	1	2	2	1	1	0	1	2	0	2	2	1	2	1	1	1	1	1	1	2	2	2	33
Held et al. (2013)^14^	1	0	0	1	0	1	1	0	1	1	0	1	1	1	0	1	1	0	0	0	0	2	1	1	15
Byrne et al. (2020)^11^	1	0	0	1	0	1	1	0	1	2	0	1	2	1	0	1	1	1	0	1	0	1	1	1	18

† Total points: 1–16 low quality, 17–32 moderate quality and 33–48 high quality^21^

### Study and patient characteristics

Overall, 389 extra-articular P1 finger fractures from a cohort of 356 patients were included (Table 3). The weighted mean age for studies which included age and sex data was 41.6 (18–93) years, with most participants being men (66.4%, 184/277). The distribution of injured digits for studies that provided fracture details was index (15.6%, 46/298), long/middle (16.3%, 49/298), ring (21.8%, 65/298) and small/little (46.3%, 138/298). A significant proportion of cases were also unstable [transverse (51.3%, 139/272), oblique (23.4%, 64/272), spiral (18.5%, 50/272) and comminuted (16.8%, 46/272)].

**Table 3:** Study characteristics.

Author (year)	Study design	Partic-ipants (n)	Fractu-res (n)	Mean age [SD] (range)	Males (%)	Finger (%)	Fracture location (%)	Fracture pattern (%)
Thomine et al. (1983)^16^	Prospective series	14	14	N.D. [N.D.] (19–69)	93%	Index (43%) Middle (29%) Ring (14%) Little (14%)	Diaphyseal (100%)	Transverse (43%) Long/oblique (43%) Comminuted (14%)
Reyes and Latta (1987)^15^	Prospective series	79	91	N.D. [N.D.] (18– N.D.)	N.D.	Not stated	Midshaft (55%) Base (45%)	N.D.
Rajesh et al. (2007)^4^	Prospective series	26	26	45 years [SD 15] (19–74)	62%	Index (12%) Middle (12%) Ring (12%) Little (64%)	N.D.	N.D.
Figl et al. (2011)^12^	Prospective series	65	78	41 years [N.D.] (18–93)	71%	Index (15%) Middle (28%) Ring (35%) Little (22%)	Proximal third (51%) Middle third (41%) Distal third (8%)	Transverse (42%) Oblique (28%) Spiral (19%) Comminuted (10%)
Franz et al. (2012)^13^	Cohort study	58	66	50 years [SD 20] (18–93)	48%	Index (19%) Middle (9%) Ring (17%) Little (55%)	N.D.	Transverse (62%) Oblique (20%) Spiral (14%) Longitudinal (4%)
Held et al. (2013)^14^	Prospective series	23	23	36 years [N.D.] (18–60)	78%	Index (22%) Middle (39%) Ring (22%) Little (17%)	Proximal third (52%) Middle third (43%) Distal third (5%)	Transverse (39%) Oblique (39%) Comminuted (22%)
Byrne et al. (2020)^11^	Prospective series	91	91	37 years [SD 17] (18–84)	69%	Index (9%) Middle (5%) Ring (18%) Little (68%)	Base (54%) Shaft (37%) Distal metaphysis (9%)	Transverse (56%) Oblique (15%) Spiral (29%) Comminuted (34%)

Note. n = number; N.D. = not described.

### Interventions

Table 4 highlights the variability in the orthotic material used, joint positioning and exercise programs.

**Table 4:** Interventions.

Author (year)	Reduction	Orthosis design	Wrist position	MCPJ position	IPJ position	Buddy strap	Time in orthosis	Exercises
Thomine et al. (1983)^16^	Displaced fractures	Plaster cast Forearm-based All digits DPC free	Forceful extension	Blocked 90° flexion	Free movement	Padded metal to all digits	5 weeks	Informal active ROM of IPJ Performed early
Reyes and Latta (1987)^15^	All fractures	Plaster cast Forearm-based All digits DPC free	30° extension	Blocked 90° flexion	Free movement	Not present	3 weeks	Informal active ROM of IPJ
Rajesh et al. (2007)^4^	All fractures with gentle reduction using boxing glove over 2-3 days	Thermoplastic MCP block orthotic Hand-based Affected digits only DPC free	Not immobilised	Blocked Maximum flexion	Free movement	Not present	3-4 weeks Extra 2-3 weeks if tender	Informal active ROM of IPJ Performed immediately Passive extension of PIPJ Vigorous physiotherapy
Figl et al. (2011)^12^	Displaced fractures	Plaster cast Forearm-based All digits DPC free	30° extension	Blocked 70-90° flexion	Free movement	Finger stall	> 4 weeks	Informal active ROM of IPJ Performed after 24 h Physiotherapy if PIPJ extension deficit
Franz et al. (2012)^13^	Displaced fractures	Group A: Plaster cast Forearm-based All digits Group B: “LuCa” cast Hand-based All digits DPC free	Group A: 30° extension Group B: Not immobilised	Group A: Immobilised 70-90° flexion Group B: Blocked 70-90° flexion	Free movement	Adjacent digits	> 4 weeks	Informal active ROM of IPJ Scheduled several times per day
Held et al. (2013)^14^	Displaced fractures	Plaster dorsal slab Forearm-based All digits DPC free	N.D.	Blocked Maximum flexion	Free movement	Adjacent digits	3 weeks	Informal active ROM of IPJ
Byrne et al. (2020)^11^	All fractures	Thermoplastic MCP block orthotic Hand-based Affected and adjacent digits	Not immobilised	Immobilised Maximum flexion	Free movement	Finger stall until 6 weeks	4 weeks	Informal active ROM of IPJ Performed immediately Scheduled hourly Light activities with buddy strap on for 2 weeks

Note. DPC = distal palmar crease; IPJ = interphalangeal joint; “LuCa” = lucerne cast; MCP = metacarpal phalangeal joint; N.D. = not described; PIPJ=proximal interphalangeal joint; ROM = range of motion

### Follow-up

The six studies^4^^,^^11^, ^12^, ^13^, ^14^^,^^16^ that reported the mean follow-up time found a weighted mean of 8.3 months (3 weeks-69 months) as detailed in Table 5.

**Table 5:** Outcomes.

Author (year)	Primary outcomes	Secondary outcomes	Complications (n)	Mean follow-up time
Thomine et al. (1983)^16^	SOFCOT: Good (43%), Fair (57%)	Mean return-to-work: 44 days	Fixed flexion deformity (25°): 1 Hyperextension (10-15°): 3 Malunion: 1 Shortening (<2 mm): 3 Ulnar deviation: 1	2.5 months
Reyes and Latta (1987)^15^	Reyes: Excellent (65%), Good (15%), Fair to Poor (20%)	N.D.	Apex palmar angulation: 1 Malunion: 1	N.D.
Rajesh et al. (2007)^4^	Belsky: Excellent (72%), Good (22%), Fair to Poor (6%) Reyes: Excellent (100%) Mean TAM: 250°	Mean return-to-work: 11.8 weeks Mean grip strength: 91.5% Mean pincer strength: 88.8%	N.D.	15 months
Figl et al. (2011)^12^	Full ROM: 86% Union: 100%	Pain: 0	Axial malalignment: 12 Extension deficit (<20°): 9 Flexion deficit (1.1 cm): 2 Swelling: 9 Weather sensitivity: 8	23 months
Franz et al. (2012)^13^	Group A: Mean TAM: 239° Mean wrist motion: 119° Group B: Mean TAM: 249° Mean wrist motion: 139°	Mean satisfaction: 9	Extension lag (5°-25°): 30 Palmar apex angulation (>2°): 30 Radial/ulnar angulation (>2°): 18 Rotational deformity (5°): 1 Group A: CRPS: 2 Group B: Loss of reduction: 2	3 months
Held et al. (2013)^14^	Acceptable reduction: 91% Union: 100%	Mean coronal angulation: 4° Mean sagittal angulation: 2°	Extension deficit (20°): 2 Extension lag (10-20°): 10 Loss of reduction: 2 Shortening (0-3 mm): 11	1.8 months
Byrne et al. (2020)^11^	Belsky: Excellent or Good (85%) Reyes: Excellent (95%), Good (5%) Mean TAM: 253°	Mean appointments required: 5.3 Median days until discharge: 43 Median pain score: 0	Loss of reduction: 3	1.8 months

Note. CRPS = Complex regional pain syndrome; n = number; N.D. = not described; ROM = range of motion; TAM = total active motion

### Outcome measures

The primary outcomes of interest were goniometrical measurements (Table 5).^4^^,^^11^, ^12^, ^13^, ^14^, ^15^, ^16^ Additional outcomes included fracture union,^4^^,^^11^, ^12^, ^13^, ^14^, ^15^, ^16^ patient satisfaction,^13^ time to return-to-work,^4^^,^^16^ grip and pinch strength^4^ and pain^11^.

The approach to assessing ROM varied from calculating TAM scores^18^ (subtracting the total extension deficits of the MCPJ and IPJ from the sum of their active flexion) to using scoring systems as detailed in Table 6.

• Studies that assessed TAM^4^^,^^11^^,^^13^ found a weighted mean TAM of 249°.
• Studies that assessed ROM using the Belsky criteria^4^^,^^11^ found ‘excellent’ and ‘good’ outcomes in 94%^4^ and 85%^11^ of patients, respectively.
• The Reyes criteria was used in three studies.^4^^,^^11^^,^^15^ Among the 29 patients in Reyes and Latta's study^15^ assigned to 6-month follow-up, 86% midshaft fractures and 39% base fractures had ‘excellent’ outcomes. The remaining 47 patients with 6-week follow-up achieved ‘excellent’ and ‘good’ outcomes in 68% and 59% of fractures, respectively.^15^ ‘Excellent’ outcomes were also found in 100% of patients in the study by Rajesh et al.^4^ and 95% in Byrne and colleagues^11^ trials.
• Thomine et al.^16^ used the SOFCOT^19^ criteria and found that 43% patients achieved ‘good’ outcomes.
• As for the studies not using a scoring system, Figl et al.^12^ found that 86% of cases returned to full ROM after conservative management. Franz et al.^13^ found no significant difference in finger TAM between the plaster and LuCa cast groups.

**Table 6:** Functional score comparison.

Author	Belsky criteria	Reyes criteria	SOFCOT criteria
**Grading**	**Excellent:** ■ TAM > 215° ■ PIPJ motion > 100° ■ Pain free ■ Nil angular or rotatory deformity ■ Nil skin abnormalities **Good:** ■ TAM > 180 ■ PIPJ motion > 80° ■ Pain free ■ Minimal angular or rotatory deformity **Poor:** ■ The remaining cases	**Excellent:** ■ Full active flexion ■ PIPJ extension < 15° **Good:** ■ Full active flexion ■ PIPJ extension 16–35 **Fair:** ■ Full active flexion ■ PIPJ extension 36–45 **Poor:** ■ Full active flexion ■ PIPJ extension > 45	**Good:** ■ Mobility > 10-80° **Fair:** ■ Mobility > 25-75° **Poor:** ■ Mobility > 30-70°
**Other findings**	N/A	■ Flexion loss > 1 cm from the tip of the finger to the palm demoted the patient to the immediate inferior group	N/A

Note. PIPJ = proximal interphalangeal joint; TAM = total active motion

Regarding secondary outcomes, it was found that all but two fractures achieved union with conservative management. Two studies reported mean return-to-work timeframes of 12 weeks^4^ and 44 days.^16^

### Complications

Reported complications included anatomical deformities such as loss of reduction, malunion, shortening or misalignment and functional abnormalities such as joint stiffness and swelling. Among the 389 total fractures, there were 50 cases^13^^,^^15^^,^^16^ of malalignment, 53 cases^12^, ^13^, ^14^ of extension deficits and 14 cases^4^^,^^16^ of shortening up to 3 mm. Seven patients required surgical intervention owing to loss of reduction.^11^^,^^13^^,^^14^ There were also two cases of complex regional pain syndrome.^13^

## Discussion

**Figure 2:**
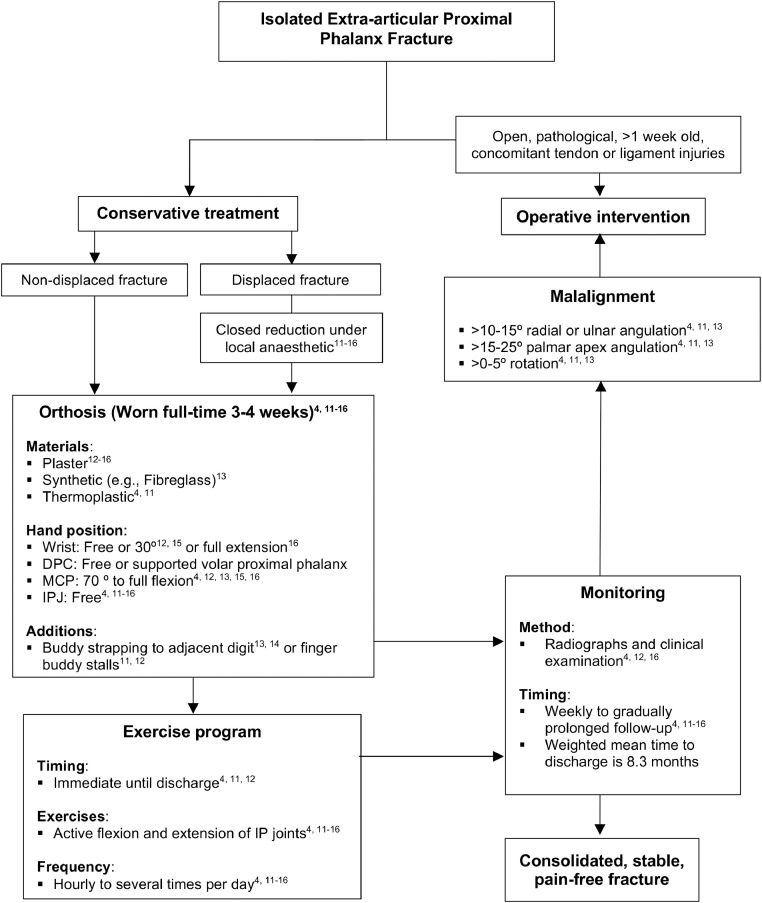
Summarised treatment protocol.

This review outlines the evidence for conservative modalities for extra-articular P1 finger fracture management, including those which are initially displaced or unstable, finding among other results that a treatment algorithm (as depicted in Figure 2) could achieve union in close to 100% (387/389) of fractures.

‘Excellent’ or ‘good’ ROM outcomes were achieved in 87% and 95% of fractures, respectively,^4^^,^^11^^,^^15^ with a weighted mean TAM of 249°,^4^^,^^11^^,^^13^ providing 96% of normal TAM.^20^ This result is also comparable to surgical fixation, highlighting how even oblique, spiral or complex fractures that are inherently unstable can be managed non-operatively provided that adequate reduction can be achieved and maintained.^21^

This is the first systematic review to the authors’ knowledge which provides a focused overview of conservatively managed extra-articular P1 fractures in adults, and supports previous studies in this domain. A recent scoping review by Vervloesem et al.^22^ in 2023 identified current methods of rehabilitation following conservative and surgical methods of management for extra-articular P1 fractures. It included 267 fractures from eight articles, five of which examined conservative interventions. All studies reported good results in mobility with a mean TAM of 240° to 258.9°, demonstrating conservative treatment as a viable alternative to surgery; similar to the mean TAM of 249° found in this review. A systematic review by Verver et al.^21^ in 2017 examined the treatment options for proximal and middle phalangeal fractures, comprising 16 studies and over 381 P1 fractures, 117 of which were managed non-operatively. It concluded that P1 fractures, even those which are initially unstable, have the potential to be treated without surgery and achieve good functional outcomes.

**Figure 3:**
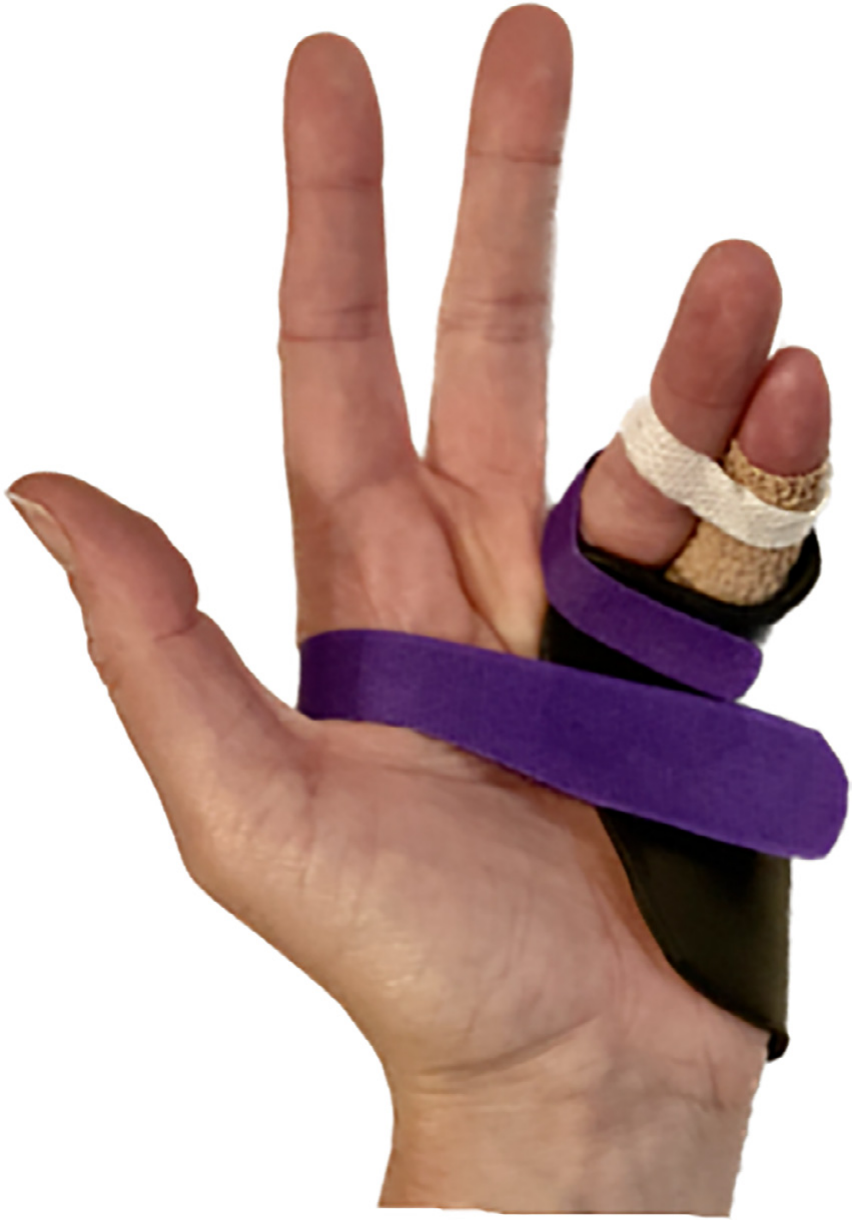
Example of custom-made thermoplastic hand-based orthosis with buddy taping produced by the authors, with permission obtained from patient for publication purposes.

The various management strategies explored in the included articles are summarised into a treatment protocol in Figure 2, with an example of a custom-made thermoplastic hand-based orthosis with buddy taping depicted in Figure 3. A variety of orthotic designs were used, with common features including the MCPJs positioned in 70-90° or maximum flexion;^4^^,^^11^^,^^14^ neighbour/buddy strapping^13^^,^^14^^,^^16^ or finger stalls^11^^,^^12^. Splint designs varied in their incorporation of all digits^12^^,^^14^, ^15^, ^16^ or only the affected and adjacent digits,^4^^,^^11^ thereby limiting unnecessary immobilisation. The IPJs were free to mobilise in all studies.^4^^,^^11^, ^12^, ^13^, ^14^, ^15^, ^16^ Using MCPJ flexion and active IPJ motion, the deforming forces of the intrinsic muscles on the P1 fragment are reduced, allowing the extensor mechanism to envelope the P1 and apply compressive forces across the fracture site.

Orthoses were either hand-^4^^,^^11^^,^^13^ or forearm-based,^13^^,^^14^^,^^16^ with one study offering a direct comparison.^13^ Franz et al.^13^ found no statistical difference in mean TAM or wrist motion at 12 weeks, suggesting potential for using less restrictive and more comfortable orthotics without compromising safety. Patients who were allowed free wrist mobilisation were noted to have increased wrist ROM initially and higher patient satisfaction.^13^

Buddy strapping^11^, ^12^, ^13^, ^14^^,^^16^ as an adjunct to orthotic intervention provides analgesia and encourages finger tracking during exercise to prevent secondary displacement. Finger buddy stalls have the added benefit of reducing oedema.^11^^,^^12^ Interestingly, paediatric studies^23^^,^^24^ have suggested buddy taping alone can be effective in treating stable fractures if used judiciously, although has yet to be studied in adults.

The material used for orthosis construction varied from plaster, synthetic casting material (e.g., fiberglass), thermoplastic and metal. Orthoses were worn full-time for three to four weeks, with some authors^4^^,^^12^^,^^13^ extending the wear time by two to three weeks if there were concerns regarding fracture healing.

All studies aimed to achieve bony healing and free mobility simultaneously by encouraging patients to actively flex and extend their IPJs as early as possible, with or without a formal hand therapy program. This allowance for free IPJ motion underpins the functional approach to conservative P1 fracture management, avoiding immobilisation and delayed motion which are the greatest predictors of poor outcome.^25^

### Clinical implications

Due to limitations of the included studies, offering conclusive recommendations should be cautioned against. This review highlights the potential to shift the management of P1 fractures, which would have been traditionally managed surgically, into the procedure room or clinic, while maintaining comparable functional outcomes.

Although the operating theatre provides certain advantages such as a controlled sterile environment and the availability of regional or general anaesthesia, it is associated with significant costs to the healthcare system and the patients and broader community. In 2012, de Putter et al.^26^ conducted a study in the Netherlands which found hand and wrist injuries to be responsible for $740 million in costs, making it the most expensive injury. $329 million was attributed to funding healthcare resources such as surgical staffing and procedural time, equipment and internal or external fixation systems,^26^ while, $411 million was attributed to lost productivity from absenteeism and reduced work capacity, largely secondary to the demographic of these injuries being men of working age, which is reflected in this review.^26^

Conservative management has demonstrably fewer complications and allows bone healing and rehabilitation to occur simultaneously,^3^^,^^4^ allowing the patient to resume work sooner^14^ and minimise the societal financial-economic burden. It also reduces the adverse consequences associated with surgery and anaesthesia such as adhesions or risk of infection. Although at present there is no study to the author's knowledge that has provided a cost-benefit analysis comparing operative fixation with conservative techniques for P1 fractures, a 2018 American study by Garon et al.^27^ on metacarpal and phalanx fractures indicated that performing closed reduction and percutaneous pinning in the procedure room reduced costs by 63.2% without an increase in complication rates. Therefore it is important, to treat P1 fractures using conservative modalities whenever it is appropriate and feasible to do so.

### Limitations

Limitations of this study are similar to those identified in two previously published reviews.^21^^,^^22^ The included articles had significant variability in study design and poor reporting quality. The orthotic design and duration of wear differed greatly between studies, often with little justification. Additionally, despite being integral to maintaining the position required for fracture alignment and healing, adherence to orthosis wear was not formally measured in any study. Finally, studies varied in their enforcement of hand therapy, and lacked detail regarding the exercises used. Significant heterogeneity existed in the criteria used to assess treatment outcomes, as well as when and how these parameters were measured. Although several studies reported goniometry measures, no uniform criteria were used. This is evidenced in studies that assessed patient outcomes using multiple scoring systems, such as in the study Rajesh et al.^4^ where all cases achieved ‘excellent’ outcomes as per Reyes's criteria, but only 72% of cases were graded ‘excellent’ according to Belsky's criteria. The variation in reporting highlighted a lack of consensus as to which parameter accurately represents meaningful results. Despite most studies including various radiological outcomes, no study assessed the significance of these findings on the quality of life or socioeconomic impact of time off work, treatment costs and lost productivity; despite functional mobility and patient satisfaction not being mutually exclusive with residual radiological or clinical deformity.^12^ The variation in follow-up time, ranging from three weeks to 69 months, also makes it difficult to draw comparisons as outcomes such as complications or treatment failure can be missed in shorter follow-up periods.

### Recommendations

There is currently a paucity of high-quality data to facilitate evidence-based treatment decision-making. Future studies would benefit from large population comparisons between surgical and non-surgical techniques, and different conservative therapies. There needs to be adequate blinding for comparison, as well as structured and transparent treatment regimens and assessment protocols. Although some comparative prospective trials have been performed, they are limited by small sample sizes^28^ or include paediatric patients^29^ which may confound the results. Future trials should assess details regarding total therapy time, rehabilitation modalities and timing, and socioeconomic costs. Qualitative surveys to elucidate the patient perspective of conservative and surgical options should be encouraged, as should the inclusion of a wider range of standardised outcomes which are more function- and patient-focused, as recommended by the International Consortium for Health Outcomes Measurement^30^ to assist in providing more holistic patient care.

Some of these suggestions are currently being addressed in a single-centre prospective trial at Austin Health in Australia by Cole et al.^31^ that is assessing the benefit of conservatively treating P1 fractures which would have conventionally been managed surgically, and a large (n = 400) multicentre randomised trial by Karantana and colleagues^32^ in the United Kingdom comparing surgical treatment with orthotic intervention.

## Conclusions

This systematic review included low-moderate level prospective case series with varying outcome measures; hence, it would be erroneous to provide definitive recommendations regarding the efficacy of conservative techniques in managing extra-articular P1 fractures in adults. Despite this, our study provides a weak recommendation that non-operative therapies for rehabilitating hand function following extra-articular P1 fractures with three to four weeks wearing an orthosis or plaster with 70-90° of MCPJ flexion, neighbour strapping, and free IPJ motion, results in close to full rates of bony union and excellent active ROM, including those with displaced or unstable fractures.

## Declaration of Competing Interests

All authors declare no financial and personal conflicts of interest.

## Appendix Supplementary materials

**Table S1.** Search Strategy performed on 17 December 2023 Image, application 1

## References

[1] van Onselen EB, Karim RB, Hage JJ, Ritt MJ. (2003). Prevalence and distribution of hand fractures. Journal of Hand Surgery (European Volume).

[2] Burkhalter WE. (1989). Closed treatment of hand fractures. The Journal of Hand Surgery.

[3] Freeland AE, Hardy MA, Singletary S. (2003). Rehabilitation for proximal phalangeal fractures. Journal of Hand Therapy: Official Journal of the American Society of Hand Therapists.

[4] Rajesh G, Ip WY, Chow SP, Fung BK. (2007). Dynamic treatment for proximal phalangeal fracture of the hand. Journal of Orthopaedic Surgery.

[5] Logters TT, Lee HH, Gehrmann S, Windolf J, Kaufmann RA. (2018). Proximal phalanx fracture management. Hand.

[6] Carpenter S, Rohde RS. (2013). Treatment of phalangeal fractures. Hand Clinics.

[7] Meals C, Meals R. (2013). Hand fractures: a review of current treatment strategies. The Journal of Hand Surgery.

[8] Page MJ, McKenzie JE, Bossuyt PM (2021). The PRISMA 2020 statement: an updated guideline for reporting systematic reviews. BMJ.

[9] Higgins J, Thomas J, Chandler J. (2019).

[10] MacDermid JC. (2004). An introduction to evidence-based practice for hand therapists. Journal of Hand Therapy.

[11] Byrne B, Jacques A, Gurfinkel R. (2020). Non-surgical management of isolated proximal phalangeal fractures with immediate mobilization. The Journal of Hand Surgery (European Volume).

[12] Figl M, Weninger P, Hofbauer M, Pezzei C, Schauer J, Leixnering M. (2011). Results of dynamic treatment of fractures of the proximal phalanx of the hand. The Journal of Trauma.

[13] Franz T, von Wartburg U, Schibli-Beer S (2012). Extra-articular fractures of the proximal phalanges of the fingers: a comparison of 2 methods of functional, conservative treatment. The Journal of Hand Surgery.

[14] Held M, Jordaan P, Laubscher M, Singer M, Solomons M. (2013). Conservative treatment of fractures of the proximal phalanx: an option even for unstable fracture patterns. Hand Surgery: An international journal devoted to hand and upper limb surgery and related research: Journal of the Asia-Pacific Federation of Societies for Surgery of the Hand.

[15] Reyes FA, Latta LL. (1987). Conservative management of difficult phalangeal fractures. Clinical Orthopaedics and Related Research.

[16] Thomine JM, Gibon Y, Bendjeddou MS, Biga N. (1983). Functional brace in the treatment of diaphyseal fractures of the proximal phalanges of the last four fingers. Annals of Hand Surgery: Official Organ of Hand Surgery Societies (Annales de Chirurgie de la Main: Organe Officiel des Sociétés de Chirurgie de la Main).

[17] Howick J, Chalmers I, Glasziou P, et al. Oxford Centre for Evidence-Based Medicine. The 2011 Oxford CEBM levels of evidence (introductory document). http://www.cebm.net/index.aspx?o=5653. [Accessibility verified December 31, 2022].

[18] Kleinert HE, Verdan C. (1983). Report of the committee on tendon injuries (International Federation of Societies for Surgery of the Hand). Journal of Hand Surgery.

[19] De la Caffiniere JY. (1981). Mansat MPost-traumatic stiffness of the long fingers. Symposium at the 55th annual meeting of SOFCOT (Raideur post-traumatique des doights longs. Symposium a la 55c reunion annuelle de la SOFCOT). Journal of Orthopedic and Trauma Surgery (Revue de Chirurgie Orthopédique et Traumatologique).

[20] Adams L, Greene L, Topoozian E. (1992).

[21] Verver D, Timmermans L, Klaassen RA, van der Vlies CH, Vos DI, Schep NWL. (2017). Treatment of extra-articular proximal and middle phalangeal fractures of the hand: a systematic review. Strategies In Trauma and Limb Reconstruction.

[22] Vervloesem N, Glassey N, Kerr A. (2023). Rehabilitation following extra-articular proximal phalangeal fractures of the fingers in adults: a scoping review. Hand Therapy.

[23] Nellans KW, Chung KC. (2013). Pediatric hand fractures. Hand Clinics.

[24] Weber DM, Seiler M, Subotic U, Kalisch M, Weil R. (2019). Buddy taping versus splint immobilization for paediatric finger fractures: a randomized controlled trial. The Journal of Hand Surgery, European volume.

[25] Rangaswamy L. (1990).

[26] de Putter CE, Selles RW, Polinder S, Panneman MJ, Hovius SE, van Beeck EF. (2012). Economic impact of hand and wrist injuries: health-care costs and productivity costs in a population-based study. The Journal of Bone and Joint Surgery. American volume..

[27] Garon MT, Massey P, Chen A, Carroll T, Nelson BG, Hollister AM. (2018). Cost and complications of percutaneous fixation of hand fractures in a procedure room versus the operating room. Hand.

[28] Khatua J, Nanda DP, Panigrahi R, Maharaj RC. (2020). Comparison of conservative and operative management for unstable extra articular proximal phalanx fracture of hand: A prospective study. Journal of Orthopaedics and Spine.

[29] Singh J, Jain K, Mruthyunjaya, Ravishankar R (2011). Outcome of closed proximal phalangeal fractures of the hand. Indian Journal of Orthopaedics.

[30] Wouters RM, Jobi-Odeneye AO, de la Torre A (2021). A standard set for outcome measurement in patients with hand and wrist conditions: Consensus by the International Consortium for Health Outcomes Measurement Hand and Wrist Working Group. The Journal of Hand Surgery.

[31] Zhang M, Cole T, Hirth M. Personal communication by email. May 18, 2022. (HREC approval HREC/83859/Austin-2022-316742v2).

[32] ISRCTN Registry. POINT: A trial of surgery versus splinting for the treatment of proximal phalanx shaft finger fractures in adults. https://www.isrctn.com/ISRCTN8826640. [Accessibility verified February 19, 2024].

