# Supplementary material for: A Systematic Review of Conservatively Managed Isolated Extra-Articular Proximal Phalanx Finger Fractures in Adults
Source: JPRAS Open. 2024 May 9;41:37–51. doi: 10.1016/j.jpra.2024.05.002 (PMC11170097; doi:10.1016/j.jpra.2024.05.002)
Supplement: Supplementary file 1 — Table S1. Search Strategy performed on 17 December 2023 [file mmc1.docx]

**Supplementary Table 1**. Search Strategy performed on 17th December 2023

| Database | Search Strategy |
| --- | --- |
| MEDLINE (n=1007) | 1.     Fracture Healing/  2.     Fractures, bone/ or fractures, closed/  3.     Fracture*.tw  4.     1 OR 2 OR 3  5.     Finger Phalanges/  6.     Finger Injuries/  7.     (finger* or phalanx*).tw  8.     5 OR 6 OR 7  9.     Conservative Treatment/  10.  Physical therapy modalities/ or exp exercise movement techniques/ or exp exercise therapy/ or exp musculoskeletal manipulations/  11.  Occupational Therapy/  12.  Orthopaedic fixation devices/ or external fixators/ or splints/ or orthotic devices/ or athletic tape/ or braces/  13.  Exp Rehabilitation/  14.  “Physical and Rehabilitation Medicine”/  15.  (Conservative OR rehabilitation OR physical therap* OR occupational therap* OR splint* OR orthotic* OR orthos* or non?surgical).tw  16.  9 OR 10 OR 11 OR 12 OR 13 OR 14 OR 15  17.  4 AND 8 AND 16  18.  Limit 17 to “humans only (removed records about animals)” |
| Embase (n=3599) | 1.     Exp fracture/  2.     Exp fracture healing/  3.     Fracture*.tw  4.     1 OR 2 OR 3  5.     Exp proximal phalanx/ or exp finger phalanx/ or exp phalanx fracture/ or exp phalanx/  6.     Exp finger fracture/ or exp hand fracture/  7.     Exp finger injury/ or finger phalanx/  8.     (finger* or phalanx*).tw  9.     5 OR 6 OR 7 OR 8  10.  Exp fracture treatment/ or exp closed fracture reduction/  11.  Exp conservative treatment/  12.  Exp physiotherapy/  13.  Exp occupational therapy/  14.  Exp dynamic exercise/  15.  Exp rehabilitation/  16.  Exp splint/  17.  Exp orthosis/  18.  Exp athletic tape/  19.  Exp brace/ or exp case brace/  20.  Exp complement fixation test/ or exp eye fixation/  21.  (conservative OR rehabilitation OR physical therap* OR physiotherapy* or occupational therap* OR split* OR orthotic* OR dynamic treatment Or tape Or brace OR orthos* OR non?surgical).tw  22.  10 OR 11 OR 12 OR 13 OR 14 OR 15 OR 16 OR 17 OR 18 OR 19 OR 20 OR 21  23.  4 AND 9 AND 22  24.  Limit 23 to human |
| Emcare (n=1129) | 1.     Exp fracture/  2.     Exp fracture healing/  3.     Fracture*.tw  4.     1 OR 2 OR 3  5.     Exp proximal phalanx/ or exp finger phalanx/ or exp phalanx fracture/ or exp phalanx/  6.     Exp finger fracture/ or exp hand fracture/  7.     Exp finger injury/ or finger phalanx/  8.     (finger* or phalanx*).tw  9.     5 OR 6 OR 7 OR 8  10.  Exp fracture treatment/ or exp closed fracture reduction/  11.  Exp conservative treatment/  12.  Exp physiotherapy/  13.  Exp occupational therapy/  14.  Exp dynamic exercise/  15.  Exp rehabilitation/  16.  Exp splint/  17.  Exp orthosis/  18.  Exp athletic tape/  19.  Exp brace/ or exp case brace/  20.  Exo complement fixation test/ or exp eye fixation/  21.  (conservative OR rehabilitation OR physical therap* OR physiotherapy* or occupational therap* OR split* OR orthotic* OR dynamic treatment Or tape Or brace OR orthos* OR non?surgical).tw  22.  10 OR 11 OR 12 OR 13 OR 14 OR 15 OR 16 OR 17 OR 18 OR 19 OR 20 OR 21  23.  4 AND 9 AND 22  24.  Limit 23 to human |
| WHO ICTRP (n=24) | Phalanx OR phalangeal OR phalanges OR finger OR fingers |
| ANZCTR (n=29) | Phalanges OR Phalangeal OR Phalanx OR Fingers OR Finger |
| ClinicalTrials.gov (n=24) | Phalanx Fracture Hand OR Finger Fracture OR Finger Injuries |
| CENTRAL (n=1164) | (Phalanx OR phalanges OR phalangeal OR finger OR fingers) AND (fracture OR fractures OR injury OR injuries) |
